# Field integration of shoot gas-exchange and leaf chlorophyll fluorescence measurements to study the long-term regulation of photosynthesis in situ

**DOI:** 10.1093/treephys/tpae162

**Published:** 2024-12-11

**Authors:** Jaakko Oivukkamäki, Juho Aalto, Erhard E Pfündel, Manqing Tian, Chao Zhang, Steffen Grebe, Yann Salmon, Teemu Hölttä, Albert Porcar-Castell

**Affiliations:** Optics of Photosynthesis Laboratory, Institute for Atmospheric and Earth System Research (INAR)/Forest Sciences, Viikki Plant Science Center, Latokartanonkaari 7-9, 00014 University of Helsinki, Helsinki, Finland; Hyytiälä Forestry Field Station, Hyytiäläntie 124, 00014 University of Helsinki, Korkeakoski, Finland; Institute for Atmospheric and Earth System Research (INAR)/Forest Sciences, Latokartanonkaari 7-9, 00014 University of Helsinki, Helsinki, Finland; Heinz Walz GmbH, Eichenring 6, D-91090 Effeltrich, Germany; Institute for Atmospheric and Earth System Research (INAR)/Forest Sciences, Latokartanonkaari 7-9, 00014 University of Helsinki, Helsinki, Finland; Optics of Photosynthesis Laboratory, Institute for Atmospheric and Earth System Research (INAR)/Forest Sciences, Viikki Plant Science Center, Latokartanonkaari 7-9, 00014 University of Helsinki, Helsinki, Finland; Optics of Photosynthesis Laboratory, Institute for Atmospheric and Earth System Research (INAR)/Forest Sciences, Viikki Plant Science Center, Latokartanonkaari 7-9, 00014 University of Helsinki, Helsinki, Finland; Institute for Atmospheric and Earth System Research (INAR)/Forest Sciences, Latokartanonkaari 7-9, 00014 University of Helsinki, Helsinki, Finland; Institute for Atmospheric and Earth System Research (INAR)/Forest Sciences, Latokartanonkaari 7-9, 00014 University of Helsinki, Helsinki, Finland; Optics of Photosynthesis Laboratory, Institute for Atmospheric and Earth System Research (INAR)/Forest Sciences, Viikki Plant Science Center, Latokartanonkaari 7-9, 00014 University of Helsinki, Helsinki, Finland

**Keywords:** alternative energy sinks, *Betula pendula*, electron transport rate, field measurements, MICRO-PAM, pulse-amplitude modulation (PAM), silver birch

## Abstract

Understanding the diurnal and seasonal regulation of photosynthesis is an essential step to quantify and model the impact of the environment on plant function. Although the dynamics of photosynthesis have been widely investigated in terms of CO_2_ exchange measurements, a more comprehensive view can be obtained when combining gas-exchange and chlorophyll fluorescence (ChlF). Until now, integrated measurements of gas-exchange and ChlF have been restricted to short-term analysis using portable infrared gas analyzer systems that include a fluorometer module. In this communication we provide a first-time demonstration of long-term, in situ and combined measurements of photosynthetic gas-exchange and ChlF. We do so by integrating a new miniature pulse amplitude modulated-fluorometer into an existing system of automated chambers to track photosynthetic gas-exchange of leaves and shoots in situ. The setup is used to track the dynamics of the light and carbon reactions of photosynthesis at a 20-min resolution in leaves of silver birch (*Betula pendula* Roth) during summertime. The potential of the method is illustrated using the ratio between electron transport and net assimilation (ETR/A_NET_), which reflects the internal electron use efficiency of photosynthesis. The setup successfully captured the diurnal patterns in the ETR/A_NET_ during summertime, including a large increase in noon ETR/A_NET_ in response to a period of high temperatures and relatively low soil moisture, pointing to a drastic decrease in electron-use efficiency. The observations emphasize the value of combined and long-term in situ measurements of ChlF and gas-exchange, opening new opportunities to investigate, model and quantify the regulation of photosynthesis in situ and the connection between ChlF and photosynthetic gas-exchange. The next steps, potential and limitations of the approach are discussed.

## Introduction

Photosynthesis, and its regulation, has been widely investigated in terms of gas-exchange measurements. These measurements are based on enclosing leaves or small shoots inside a cuvette and feeding it with air of known chemical composition. Subsequently, infrared gas analyzers (IRGA) are used to analyze the chemical composition of the air flowing out of the cuvette. Differences in the concentration of CO_2_ between the incoming and outgoing air provide information on the net exchange of CO_2_ (A_NET_) as mediated by the rate of RuBisCO carboxylation (true photosynthesis), RuBisCO oxygenation (photorespiration) and mitochondrial respiration (Farquhar et al. 1980, Von Caemmerer and Farquhar 1981, Lawson et al. 2014). Likewise, changes in the concentration of H_2_O provide information on the rate of transpiration and can be used to estimate the dynamics of stomatal conductance (*g_s_*) and, by extension, the concentration of CO_2_ in the intercellular air spaces, an essential parameter to model photosynthesis (Von Caemmerer and Farquhar 1981, Flexas et al. 2002, Flexas et al. 2004). As such, portable IRGA systems have been widely used to measure and model the environmental regulation of photosynthesis, either by following the dynamics of A_NET_ under natural conditions, or by investigating the response of A_NET_ to changes in irradiance (Björkman 1981, Demmig-Adams and Adams III 1992), temperature (Bernacchi et al. 2001, Sage and Kubien 2007) or CO_2_ availability (Cornic and Briantais 1991, Ainsworth and Rogers 2007). Similarly, long-term automated chamber systems can be deployed in the field to investigate the diurnal and seasonal regulation of photosynthesis in situ (Bloom et al. 1980, Hari et al. 1999, Kolari et al. 2014). Although highly informative, measurements of photosynthetic CO_2_ exchange provide only a one-sided view of the photosynthetic process from the perspective of the carbon reactions, but the regulation of photosynthesis can be also approached from the point of view of the light reactions (Kramer et al. 2004, Gu et al. 2019).

Chlorophyll fluorescence (ChlF) has been used extensively to investigate the regulation of the light reactions of photosynthesis (Genty et al. 1989, Maxwell and Johnson 2000, Murchie and Lawson 2013). For example, ChlF can be used to estimate the photochemical efficiency of photosystem II (PSII) and the rate of linear electron transport between PSII and PSI (ETR) which is coupled to the production of ATP and NADPH by the light reactions (Genty et al. 1989, Klughammer and Schreiber 1994), in addition to multiple other parameters tracking the regulation of photochemical and non-photochemical processes in the photosystem, as reviewed by Lazár (2015). Overall, although ETR can be potentially inferred from gas-exchange measurements (Harley et al. 1992, Loreto et al. 1994), the combination of independent measurements of ChlF and gas-exchange provide a more complete view of the regulation of photosynthesis (Cornic and Briantais 1991, Laisk and Loreto 1996, Yin et al. 2011) that can be used to investigate how the production of NADPH is partitioned between alternative pathways and RuBisCO carboxylation/oxygenation reactions (Laisk and Loreto 1996, Morfopoulos et al. 2014), to study the dynamics of mesophyll conductance (*g_m_*) (Harley et al. 1992, van der Putten et al. 2018) or to estimate the rate of leaf respiration in the light (Yin et al. 2009). For example, the ratio between ChlF-based ETR and net CO_2_ assimilation (ETR/A_NET_) provides information on the efficiency of energy conversion between light and carbon reactions of photosynthesis, as affected by the dynamics of mitochondrial respiration, alternative electron sinks or photorespiration (see recent review by Perera-Castro and Flexas 2023), responding to multiple stress factors, such as drought (Flexas et al. 2002) or nutrient imbalances (Bazihizina et al. 2015).

Clearly, the combination of ChlF and gas-exchange is essential to investigate and model the environmental regulation of photosynthesis, and the connection between ChlF, now measurable across spatial and temporal scales as solar-induced ChlF or SIF (Porcar-Castell et al. 2021), and photosynthetic gas-exchange.

Commercially available portable photosynthetic IRGA systems provide an option for combining gas-exchange and ChlF measurements. However, these systems are not suitable for continuous, unattended and long-term operation in the field. Likewise, although in situ and long-term automated photosynthetic gas-exchange (Bloom et al. 1980, Kolari et al. 2014) and pulse amplitude modulated (PAM) ChlF systems (Porcar-Castell et al. 2008, Porcar-Castell 2011, Martini et al. 2022) have been separately available for quite some time, their long-term integration is difficult and has not yet been accomplished or demonstrated. This limitation could now be overcome with the introduction of the MICRO-PAM (Heinz Walz GmbH, Effeltrich, Germany) system, a miniaturized and weatherproof PAM fluorometer for long-term field operation.

We here present a first demonstration of integrated, continuous and long-term PAM-ChlF and gas-exchange measurements for application in field conditions. The new MICRO-PAM was coupled to an automated chamber system for combined and long-term measurements of ChlF and photosynthetic gas-exchange. The setup was used to follow the regulation of photosynthesis, including ETR/A_NET_, in leaves of silver birch (*Betula pendula* Roth) growing in the top-canopy in a boreal forest during summer 2021. The potential of the measurements is demonstrated by examining and discussing the diurnal patterns of ETR/A_NET_ and its response to high temperatures and water stress during summer.

## Materials and methods

**Figure 1:**
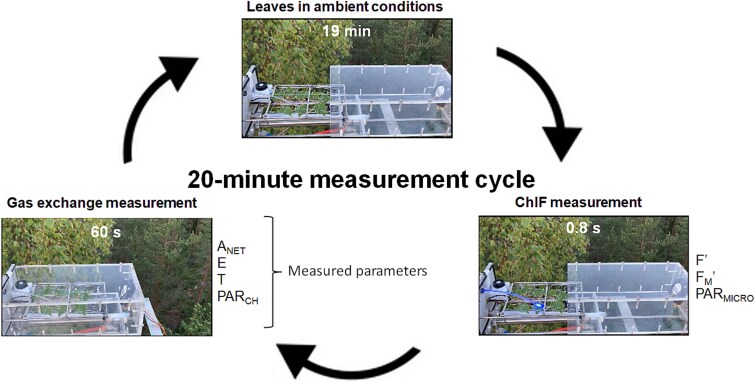
The measurement setup and the 20-min cycle for the integrated gas-exchange and ChlF measurements. Parameters recorded during the cycle are indicated in the diagram and include current PAM fluorescence yield (F′), maximal fluorescence yield (F_M_′), PAR from the MICRO-PAM sensor (PAR_MICRO_), net CO_2_ assimilation (A_NET_), transpiration rate (*E*), as well as chamber air temperature (*T*) and PAR (PAR_CH_). See also video material in the Supplementary data.

Measurements were conducted in the top canopy of a 60-year-old silver birch located at the Station for Measuring Forest-Ecosystem-Atmosphere Relations (SMEAR) II station (Hari et al. 2013) in Southern Finland (61°51′ N, 24°17′ E, and 180 m of elevation), between 29 June 2021 and 9 August 2021. This measurement period included two periods of high temperatures, 13–14 July and 26–27 July, hereafter called as high-temperature periods. Top canopy foliage was accessed from permanently installed scaffolding towers. Two adjacent and south/south-west facing shoots were selected from the top of the tree and separately used to: (i) install the autonomous shoot gas-exchange chamber and MICRO-PAM fluorometer (Figure 1) and (ii) conduct benchmarking measurements using a MONITORING-PAM (hereafter referred to as MONI-PAM) fluorometer (Heinz Walz GmbH). Due to a power failure, we had a 5-day gap in data between 30 July and 4 August.

### Long-term measurements of shoot-level gas-exchange

The shoot chamber used for gas-exchange measurements was made of acrylic plastic and had a volume of 2.1 dm^3^. The opening and closing of the chamber was activated with a pneumatically operated system (Figure 1 and Supplementary Video, available as Supplementary data at *Tree Physiology* Online) that operated at a frequency of c. 20 min, allowing for regular, automated and long-term measurements of CO_2_ and H_2_O fluxes. The frequency of the measurement cycle is constrained by the total number of chambers connected to the same IRGA analyzer and also by the frequency of the saturating pulses required for ChlF measurements, which should not be too high to avoid long-term damage. Additionally, the chamber housed a PAR sensor (LI-190/R, Li-Cor Inc., Lincoln, NE, USA) situated at the base of the chamber and a t-type thermocouple that was placed underneath the shoot to measure chamber temperature. The chamber was closed for 60 s every 20 min. When closed, chamber air was sampled at ~1 L per min via a perforated fluorinated ethylene propylene tubing placed below the shoot. The chamber closure is non-hermetic, which allows for air inflow into the chamber during gas sampling. A fan installed inside the chamber turned on during chamber closure to ensure proper air mixing. The concentration of CO_2_ and H_2_O in the sampled air was monitored at 5-s intervals with an IRGA system (LI-840A, Li-Cor Inc.) located in a nearby cottage. Chamber air temperature measurements were likewise conducted during chamber closure at 5-s intervals. The chamber enclosed eight leaves arranged on a 2D plane with the help of transparent fishing lines to retain the leaves in a stable position. Total leaf area (42.5 cm^2^) was estimated using image processing software (ImageJ, Schneider et al. 2012). Subsequently, the net rate of uptake/emission of CO_2_ or transpiration of H_2_O per leaf surface area was calculated by analyzing the rate of increase/decrease in the concentration of the gas inside the chamber during the closure period. For a more detailed explanation of the shoot chamber system, see Hari et al. (1999).

### Long-term measurements of ChlF with the MICRO-PAM and MONI-PAM systems

A MICRO-PAM fluorometer (Heinz Walz GmbH) was installed in the chamber, allowing for simultaneous measurements of fluorescence parameters. The MICRO-PAM fluorometer is relatively small (13.5 cm × 4 cm × 3.5 cm) and weatherproof, facilitating the integration with a field gas-exchange system and the long-term operation. The MICRO-PAM unit of this study used blue measuring light and saturating pulses as excitation source and houses a longpass filter (50% transmittance at 645 nm) for ChlF measurements. Measuring light, saturating pulses and ChlF are transmitted through a 4.6 cm light guide. In the present study, this light guide was replaced by a longer and curved, 10-cm version to facilitate the measurement of a leaf inside the chamber with the MICRO-PAM attached to the back of the chamber (Figure 1). The tip of the light guide was placed ~3–5 mm above the leaf surface at a 35° angle from the leaf plane, measuring an approximate area of 1 cm^2^. The duration of the saturating pulse was set to 0.8 s and the intensity to maximum, which delivered ~6700 μmol m^−2^ s^−1^ of PAR. The intensity of the measuring light was ~1 μmol m^−2^ s^−1^. See also video in Supplementary data for a closer view of the combined ChlF and gas-exchange setup.

The MICRO-PAM system includes a thermocouple for contact temperature measurements and a quantum light sensor for PAR measurements mounted on a leaf clip. As chamber air temperature and PAR variables were already being measured by separate sensors inside the shoot chamber, and since we did not require the leaf clip, the sensors as well as the leaf clip were detached from the MICRO-PAM. The PAR sensor (PAR_MICRO_) was moved to the top of the chamber, back frame and used for quality control during post processing. The thermocouple was not used in this study but could be placed inside the chamber in the future to provide additional temperature measurements.

Both the MICRO-PAM and MONI-PAM fluorometers were operated through a MONI-DA data acquisition and control unit (Heinz Walz GmbH), housing a micro-SD flash memory card for data storage. The MONI-DA unit can operate multiple PAM fluorometers either using a clock-function or via pre-programmed batch files. In this study, we used the clock function to trigger measurements at a frequency of 20 min, to match that of the gas-exchange measurements. Originally, the MONI-DA was set to trigger ChlF measurements a few seconds before chamber closure, although the synchronization was lost after a few days (see below). Every 20 min, both the MICRO-PAM and MONI-PAM fluorometers registered the current and maximal PAM fluorescence yields, F′ and F_M_′, respectively, allowing for the estimation of the quantum yield of PSII Y(II), the rate of liner electron transport (ETR) and—during nighttime—the maximum quantum yield of PSII or F_V_/F_M_ (see section: Data quality control).

### Reference measurements and micrometeorological data

A MONI-PAM system installed in a nearby shoot was used as reference. Similar to the MICRO-PAM, the MONI-PAM measures F′ and F_M_′, using a blue measuring light and saturating light pulses (Porcar-Castell et al. 2008). The main differences between the devices are in the geometry of the leaf clip as well as in the fore optics and PAR and temperature measurements. In the MONI-PAM, the leaf is attached with a clip c. 25 mm away from the measuring light, preventing movement of the sample area. It is important to note that despite of the differences in fore optics, the saturating pulse graphs provided by the WinControl software for diagnostic purposes were not indicative of undersaturation in neither the MONI-PAM nor MICRO-PAM setups, with F_M_ levels being reached within the pulse duration (see Figure S5 available as Supplementary data at *Tree Physiology* Online).

Additional micrometeorological data was obtained from the background measurements at SMEAR-II Station. Atmospheric pressure was recorded from a nearby barometer (Druck DPI 260, Baker Hughes, Houston, TX, USA) at ground level, while precipitation data was gathered using a weather sensor (Vaisala FD12P, Vaisala, Vantaa, Finland) located in a nearby tower at 18-m height. Ambient temperature data (used to assess micrometeorological conditions) was collected with a PT-100 platinum resistance thermometer also located in a nearby tower. Additionally, soil water content data was collected using soil moisture sensors (Delta-T ML3, Delta-T Devices Ltd, Cambridge, UK), averaged from five locations near the measurement tower.

### Data analysis and processing

In addition to direct measurements of A_NET_ and E, we estimated saturation vapor pressure (SVP), vapor pressure (VP), vapor pressure deficit (VPD) and stomatal conductance (*g_s_*) on the basis of chamber air temperature and atmospheric pressure. The SVP (kPa) was first calculated using the Magnus–Tetens equation (Tetens 1930, Monteith et al. 1994):


(1) \begin{equation*} \mathrm{SVP}=0.61078\ \exp \frac{17.27\times T}{T+237.3} \end{equation*}

where *T* is the air temperature inside the chamber (°C). Next, we calculated vapor pressure of the air, VP (kPa), following the ideal gas law:


(2) \begin{equation*} \mathrm{VP}={}^{n}\!/{}_ {V}\times \left(R\times \left(T+273.15\right)\right) \end{equation*}

where *n*/*V* is the measured concentration of H_2_O in the air (mol m^−3^), and *R* is the ideal gas constant (in J mol K^−1^). Subsequently, VPD (kPa) was estimated as:


(3) \begin{equation*} \mathrm{VPD}=\mathrm{SVP}-\mathrm{VP} \end{equation*}

Finally, VPD was used in the calculation of stomatal conductance (*g_s_*) (mmol m^−2^ s^−1^), as:


(4) \begin{equation*} {g}_s=\frac{E}{\mathrm{VPD}}\times P \end{equation*}

where *E* stands for the transpiration rate (mmol m^−2^ s^−1^) measured by the gas-exchange system and *P* for the atmospheric pressure (kPa). It is important to note that we did not measure leaf temperature, which could be a few degrees different than air temperature and should be used instead of *T* in Eq. (1) for accurate estimation of VPD and *g_s_*. Although values of VPD and *g_s_* were here used only for illustrative purposes we acknowledge that VPD could be slightly underestimated (and *g_s_* overestimated) if leaf temperature increases above chamber air temperature.

To calculate the rate of linear electron transport (ETR), we first calculated the quantum yield of PSII (Y(II)), as:


(5) \begin{equation*} Y(II)=1-\frac{F^{\prime }}{{F_M}^{\prime }} \end{equation*}

And subsequently estimated ETR as:


(6) \begin{equation*} \mathrm{ETR}={\mathrm{PAR}}_{\mathrm{CH}}\times Y\kern-1pt\left(\mathrm{II}\right)\times \mathrm{Abs}\times{\alpha}_{\mathrm{II}} \end{equation*}

where PAR_CH_ is the photosynthetically active radiation recorded by the chamber sensor (μmol m^−2^ s^−1^), Abs is the PAR absorption by the leaf and α_II_ is a coefficient that accounts for the fraction of radiation absorbed by PSII antenna. As a first approximation, we here use the generalized values for Abs and α_II_ of 0.84 and 0.5, respectively (Maxwell and Johnson 2000). Yet, despite that the average PAR absorption of silver birch leaves, measured with an integrating sphere in a nearby experiment, was indeed very close to the standard 0.84 value during the growing season (Atherton et al. 2017), it should be emphasized that leaf PAR absorption and specially α_II_ may depart from these standard values or present seasonal variation. Complementary measurements of these parameters are therefore highly recommended for accurate estimation of ETR.

Maximum levels of nighttime (i.e., dark-acclimated) Y(II) were here taken as an estimate of the maximum quantum yield of PSII, or F_V_/F_M_, where F_V_ stands for variable fluorescence (F_V_ = maximal fluorescence (F_M_) − minimum fluorescence (F_0_)).

Finally, we estimated the ratio ETR/A_NET_ as a simple means to evaluate and demonstrate the value of the combined ChlF and gas-exchange measurements, without need to go into a full analysis of photosynthetic energy partitioning and invoke further assumptions to estimate day respiration, mesophyll conductance and photorespiration.

### Data quality control

Although the MICRO-PAM and gas-exchange measurements were synchronized at the start of the measurements, the clock of the MONI-DA unit drifted and the measurements got gradually out of sync after a few days. This drift produced a temporal mismatch between the two measurements of up to 8 min. While the effect of this mismatch is expected to be minor if the illumination remains stable during this period, it could lead to discrepancies between the gas-exchange and MICRO-PAM data under conditions with varying illumination (e.g., cloudy days). To identify and exclude measurement pairs (i.e., ChlF and gas-exchange data) registered under contrasting PAR levels, we calculated the ratio of PAR measured by the chamber quantum sensor (PAR_CH_) and PAR measured by the MICRO-PAM quantum sensor (PAR_MICRO_), which reflected the illumination conditions at the time of gas-exchange and PAM-ChlF measurements, respectively. Despite that the MICRO-PAM quantum sensor was positioned at the back of the chamber, differences in the ratio PAR_CH_/PAR_MICRO_ can still be used to discriminate measurements collected under diverging light environment. Subsequently, measuring points where the ratio was outside the values of 0.5–2.0 (see Figure S1 available as Supplementary data at *Tree Physiology* Online) were filtered out and excluded from the analysis. A stricter filter using a range of 0.75–1.5 did not affect the patterns in the observations (data not shown) so we decided to stick to the less strict 0.5–2.0 for better illustration.

## Results and discussion

### The environmental response of net CO_2_ assimilation and electron transport rate: diurnal and long-term patterns.

**Figure 2:**
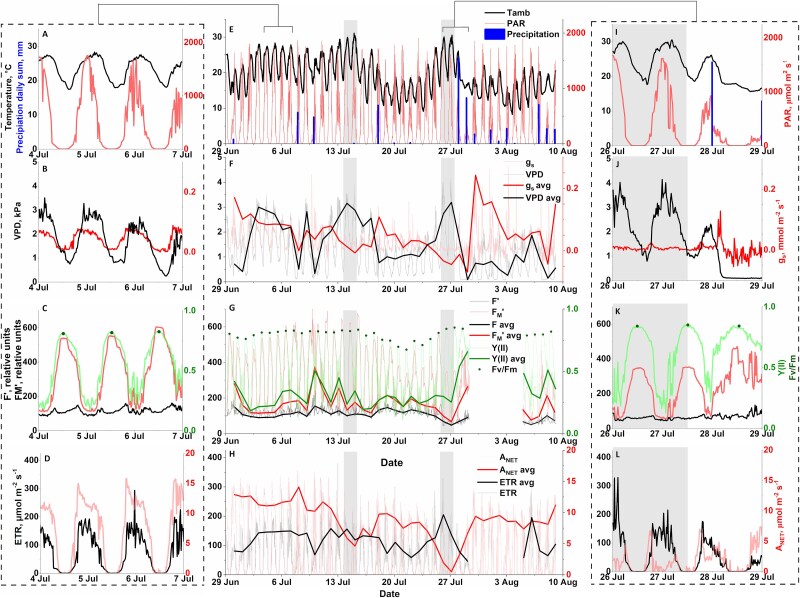
Time series of air temperature, PAR and precipitation (A, E, I); VPD and stomatal conductance (B, F, J); quantum yield of PSII Y(II), maximum quantum yield of PSII or F_V_/F_M_, current (F′) and maximal PAM fluorescence yield (F_M_′) (C, G, K); as well as the rates of linear electron transport (ETR) and net CO_2_ assimilation (A_NET_) (D, H, L), throughout the study period. The left column panels provide a highlight of early July measurements, while the right column panels highlight the second high temperature period. Thick lines in the middle panels denote noon averages (11:00–15:00 h). Grey shading in middle and right-hand columns indicate high temperature periods. The date label presented on the x-axis is situated at noon for each corresponding date.

Diurnal patterns of variation in chamber PAR and temperature were typical of the boreal summer (Figure 2A), with very short nights, radiation levels of up to 1700 μmol m^−2^ s^−1^ during daytime and daily temperature fluctuations of 10–15 °C. Diurnal temperature fluctuations were also reflected in the daily pattern of VPD and, by extension, in leaf stomatal conductance (*g_s_*) (Figure 2B). During sunny days, stomatal conductance started to increase at sunrise in response to PAR, with maximum *g_s_* levels before noon, and gradually decreasing towards the afternoon with increasing VPD (but note how this pattern was muted during the second high temperature period in late July, Figure 2J).

In turn, the diurnal patterns in the quantum yield of PSII (Y(II)) (Figure 2C), linear electron transport rate (ETR) and net assimilation (A_NET_) (Figure 2D), as acquired with our integrated measurements, not only captured the direct influence of PAR and temperature on ETR and A_NET_, but highlighted also the contrasting regulatory dynamics that exist between light and carbon reactions throughout the day. Although both ETR and A_NET_ increased during the morning and decreased towards the evening in response to PAR, the daily patterns of variation were clearly different, with ETR peaking around noontime, whereas maximum A_NET_ was observed in the morning hours, when stomatal conductance was higher (Figure 2C and D). Daytime A_NET_ values in the beginning of July were around 15 μmol m^−2^ s^−1^, consistent with previous observations for birch in the area (Aalto and Juurola 2001, Atherton et al. 2017). Finally, the daily variation in Y(II), decreasing around noontime with increasing PAR levels, was consistent with the known activation of regulated thermal dissipation or non-photochemical quenching (NPQ) (Porcar-Castell 2011), although photochemical quenching (PQ) dynamics could also contribute to the variation (data not shown).

Throughout the study period, the dynamics in air temperature, PAR and precipitation were indicative of a relatively hot summer (Figure 2E). The mean temperature for July 2021 was 19.0 °C, noticeably higher than the July mean temperature of 16.2 °C for the period 1991–2020 in Hyytiälä (Jokinen et al. 2021). Interestingly, there were two periods when daily temperatures surpassed 30 °C (14–15 July and 26–27 July), which were here used to further emphasize the informative value of our integrated measurements (highlighted with grey in Figure 2). Furthermore, although the amount of precipitation for July was within normal levels, with 97 mm compared with an average of 85 mm for the period 1991–2020 (Jokinen et al. 2021), precipitation was concentrated towards the end of the study period.

Together, the combination of high temperatures (promoting increased transpiration) and limited precipitation resulted in a gradual decrease in soil water content during the study period (see Figure S2 available as Supplementary data at *Tree Physiology* Online), which reached minimum levels during the second high temperature period, just before precipitation partly replenished the soil water storage. These dynamics are consistent with the gradual decrease observed in the daily average *g_s_* from the beginning of the study period until the rainy day on the 28 July (Figure 2E), denoting the stomatal response to the increasingly limited plant water availability and high evaporative demand (Figure 2F). The regulatory response to decreasing plant water status could be best appreciated during the second high temperature period, where VPD levels of up to 4 kPa combined with minimal soil water availability resulted in *g_s_* values close to zero (Figure 2F and G), which is a typical response to water stress, as the plant tries to conserve water by keeping the stomata relatively closed throughout the day (Martin-StPaul et al. 2017).

Similar to *g_s_*, the daily average A_NET_ gradually decreased during July, and especially during the second high temperature period (Figure 2L), when net assimilation around noontime was close to zero and likely limited by low stomatal conductance and CO_2_ availability. Decreased stomatal conductance would reduce the concentration of CO_2_ inside the leaf, promoting the oxygenation of RuBisCO (photorespiration) relative to the carboxylation (true photosynthesis) (Farquhar and Sharkey 1982, Flexas et al. 2000). In addition, increasing temperature also improves the affinity of RuBisCO for O_2_, further enhancing photorespiration (Ogren 1984, Flexas et al. 2002), and increases the rate of mitochondrial respiration (Busch 2020), which could have further contributed to decrease in A_NET_ during high temperature periods.

Unlike A_NET_ and *g_s_*, the diurnal and seasonal patterns in Y(II) and ETR remained relatively stable during the study period and did not present a clear response to the decreasing plant water status. This is consistent with previous observations indicating that ETR and PSII activity is not as severely affected by drought as net CO_2_ assimilation (Flexas et al. 1998, 2002, Lu and Zhang 1998, Helm et al. 2020).

A stable seasonal pattern could be also noted in terms of maximal quantum efficiency of photochemistry (F_V_/F_M_), which decreased only in response to the cooler days between the two high temperature periods, but not in response to water limitations. Similar dynamics were registered by the reference MONI-PAM dataset (see Figure S4 available as Supplementary data at *Tree Physiology* Online). The limited response of F_V_/F_M_ to water stress and its high sensitivity to low temperatures have been described earlier (Wang and Huang 2004, Oliveira and Peñuelas 2005). In turn, the decrease of F_V_/F_M_ during the cooler days could be associated with the accumulation of sustained forms of regulatory non-photochemical quenching or NPQ in response to high irradiance and lower temperatures (Porcar-Castell 2011, Verhoeven 2014), which would be consistent with the observed decrease in F_M_′ for the same period (Figure 2G). Overall, the results emphasize the added informative potential of field integrated ChlF and gas-exchange measurements whereby we can gain deeper insight into the environmental response of photosynthesis in situ by simultaneously following the dynamics of light and carbon reactions.

Noticeably, we observed a sudden decrease in the level of current (F′) and maximal (F_M_′) PAM fluorescence yields recorded by the MICRO-PAM system that took place between two consecutive measuring points during the hot morning of the 26th of July at the start of the second high temperature period (Figures 2G and S3A, available as Supplementary data at *Tree Physiology* Online). The decrease did not reverse and levels of F′ and F_M_′ remained lower for the rest of the study period. Such a decrease was however not registered in the reference MONI-PAM dataset, neither associated with a clear change in the quantum yield of PSII (see Figure S3A available as Supplementary data at *Tree Physiology* Online), which would point to a technical reason. Since the decrease was not gradual it could suggest that there was a movement of the leaf being measured, decreasing the absolute PAM signal levels. Leaves were kept in place by transparent fishing lines, and it is possible that these lines got loose in response to the high temperature, allowing the leaf to move slightly off the fiber field of view. Yet, we did not observe any significant differences in the slope of the relationship between the nighttime F_V_/F_M_ levels from the MICRO-PAM and MONI-PAM systems when comparing the days prior and posterior to this sudden change (see Figure S3B available as Supplementary data at *Tree Physiology* Online), suggesting that the decrease in F′ and F_M_′ had not disrupted the capacity of the MICRO-PAM to estimate the quantum yield of PSII, and by extension ETR. We did however observe a systematic difference in F_V_/F_M_ levels between the two instruments, where MONI-PAM F_V_/F_M_ levels tended to be slightly higher and presenting lower sensitivity to the cooler nights (see Figure S3B available as Supplementary data at *Tree Physiology* Online). We are unsure as to what could be the cause of these differences but given that both systems used the same blue measuring light, they could be related to the different measurement geometry, calibrations, differences in the local light environment between leaves or differences in the intensity of the saturating pulses between the two systems, although the shape of the SP curves did not show signs of lack of saturation (see Figure S5 available as Supplementary data at *Tree Physiology* Online).

### The potential of in situ and integrated measurements of ChlF and gas-exchange: the case of the ETR/A_NET_ ratio

The potential of our integrated ChlF and gas-exchange measurement setup was further demonstrated by resolving the diurnal and long-term dynamics in the ETR/A_NET_ ratio. The ETR/A_NET_ ratio reflects the internal efficiency of energy conversion between light and carbon reactions of photosynthesis, denoting the number of electrons that are being transported for every molecule of CO_2_ contributing to the net CO_2_ balance of the leaf (i.e., net photosynthesis). The theoretical minimum value for the ETR/A_NET_ is 4, since four electrons are needed for the generation of the two NADPH molecules that are required for the fixation of a CO_2_ molecule by the Calvin–Benson–Bassham cycle (Farquhar et al. 1980). Yet, processes, such as photorespiration, mitochondrial respiration or alternative energy sinks, can decrease the internal efficiency (Alric and Johnson 2017, Walker et al. 2020) and values between 8 and 10 are normal for non-stressed C_3_ plants (Perera-Castro and Flexas 2023).

**Figure 3:**
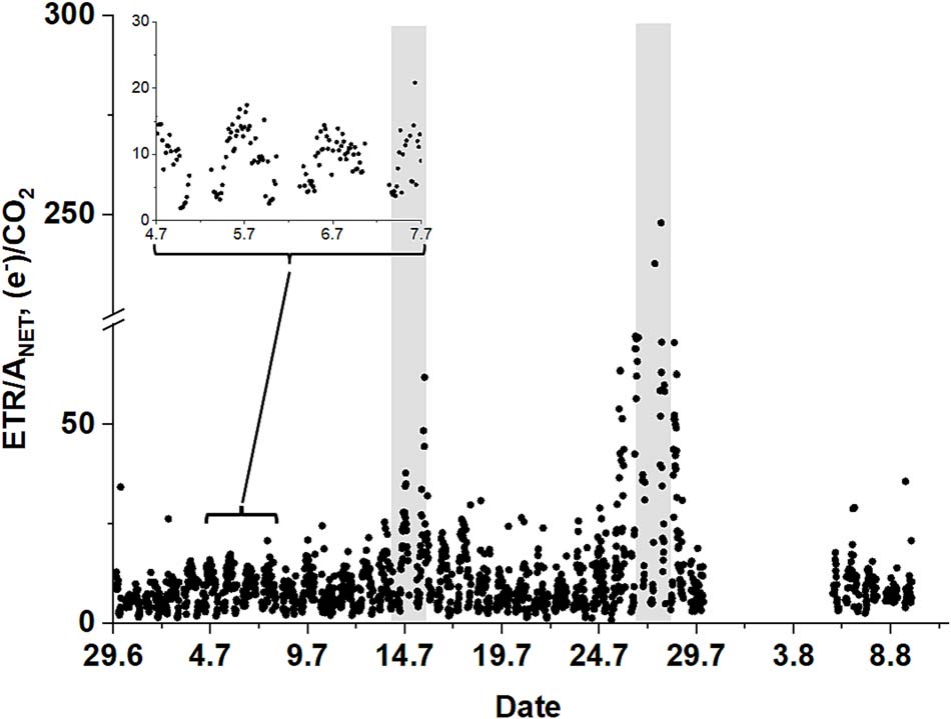
Time series of the ETR/A_NET_ ratio in birch leaves obtained with our integrated ChlF and gas-exchange measuring system during the study period. The two high temperature periods are highlighted with grey shading. An example of the diurnal variation can be seen in the inset for illustrative purpose. Note how ETR/A_NET_ values increase around noon and especially in response to the high temperature periods.

To the best of our knowledge the method here reported provides a first-time illustration of the in situ and long-term variation in the ETR/A_NET_ ratio, obtained here at a high temporal resolution of 20 min. During early July (Figure 3, inset), the diurnal variation ranged from 3.2–5 at dawn and during sunset, to ~14–17 electrons per net CO_2_ assimilated around noon, which is when the ratio peaked. These values are similar to earlier studies done on *Betula ermanii* and *Betula platyphylla*, where ETR/A_NET_ values of ~11 and 9 were presented, respectively (Kitao et al. 2003, 2015). Likewise, the ETR/A_NET_ ratio increased to values of up to 62 and 247, during the first and second high temperature period, respectively, before returning to normal levels (Figure 3). On one hand, these diurnal and seasonal patterns would be largely consistent with the contrasting dynamics in Y(II), ETR and A_NET_ discussed above, reflecting the increased role of autotrophic respiration and photorespiration around noon-time as well as during the high temperature periods, whereby decreasing stomatal conductance, limiting CO_2_ diffusion into the chloroplasts, together with increasing temperature would increase the respiratory rates and shift the partitioning of electrons (via NADPH) from Rubisco carboxylation to its oxygenation (Flexas et al. 2002, D’Ambrosio et al. 2006). On the other hand, the diurnal and seasonal dynamics in the ETR/A_NET_ ratio can also pinpoint multiple other physiological and methodological factors.

Physiologically, in addition to the dynamics of autotrophic respiration or stomatal conductance, changes in mesophyll conductance (Harley et al. 1992, van der Putten et al. 2018), alternative electron sinks (Laisk and Loreto 1996, Alric and Johnson 2017), changes in PAR absorption mediated by chlorophyll changes or chloroplast movements (Blache et al. 2011, Pfündel et al. 2018) or dynamics in the distribution of absorbed excitation energy between PSII and PSI via α_II_ could all contribute to the diurnal and seasonal patterns of ETR/A_NET_ as shown in Figure 3. In other words, the integrated ChlF and gas-exchange measurements reported in this study can provide a valuable source of data that can be used to investigate the dynamics and the functional significance of these processes in situ, test hypothesis and eventually improve our capacity to model photosynthesis.

Methodologically, ETR/A_NET_ values during the early morning and late evening hours were clearly below the theoretical minimum. This bias would suggest that our measurements were likely underestimating ETR. Possible causes for ETR underestimation could be related to limitations in the cosine response of the PAR sensor, which could have underestimated PAR at low solar elevation, as well as differences in the footprint of ChlF and gas-exchange measurements, where ChlF measurements are conducted in a single leaf while gas-exchange measurements are carried out at the shoot level including several leaves as well as the stem therein. Likewise, the ChlF measurements were here conducted using a first version of the MICRO-PAM that used blue measuring light. Blue light is mostly absorbed by the top layers of chloroplasts in the leaf palisade (Vogelmann and Han 2000), which are exposed to higher radiation levels and may therefore present lower quantum yields, compared with chloroplasts located deeper in the spongy mesophyll. In contrast, gas-exchange measurements provide an integrated measure of photosynthetic gas-exchange across all chloroplast layers, which could contribute to the underestimation of ETR/A_NET_ levels. MICRO-PAM versions with red or amber measuring light (currently available) might prove useful to improve the method. Last but not least, it is also important to keep in mind the mismatch in the temporal response of PAM ChlF measurements and gas-exchange to variations in the environment, with ChlF responding at time scales of nanoseconds (and typically recorded at >10 Hz frequencies), and gas-exchange responding and being recorded at time scales of a few seconds. In this study, we assumed both ChlF and gas-exchange were at steady state during measurements.

Furthermore, in the absence of data and given the demonstrative purposes of this technical note, we did not correct our F-levels for the contribution of PSI, which can contribute with around 30% to the minimal F_0_ PAM levels in C_3_ plants (Pfündel 1998). If uncorrected, the contribution of PSI fluorescence will result in underestimation to the quantum yield of PSII (Pfündel 1998, Porcar-Castell et al. 2006), which could also contribute to the observed underestimation in ETR/A_NET_. Similarly, we used a fixed standard level for PAR absorption (*Abs*) and the fraction of absorbed light allocated to PSII units (α_II_) in Eq. (6), which could also affect the accuracy of our ETR/A_NET_ estimates. Overall, although methods for the estimation of PSI contribution, *Abs* and α_II_ do exist (Genty et al. 1990, Valentini et al. 1995, Pfündel 1998, Yin et al. 2009) they are very time consuming and seldom considered in the quantitatively and joint assessment of ChlF and photosynthetic gas-exchange. Clearly, versatile methods to continuously track the dynamics of these factors in the field are still need to be developed, which could help to improve the interpretation of the data. Last but not least, we cannot rule out the possibility that the saturating pulses of the MICRO-PAM would not have completely saturated the electron transport chain so that the F_M_′ values would have been underestimated and by extension the quantum yields and ETR. Although the pulse graphs do not point to this limitation (see Figure S5 available as Supplementary data at *Tree Physiology* Online), this factor would require further assessment. Possible options to correct for this undersaturation could include the use of the multiphase flash method (Loriaux et al. 2013), or the installation of the fiber with a higher angle of incidence respective to the leaf for increased radiation and signal. It is important to note that the high levels of ETR/A_NET_ reported here during the second high temperature period cannot be explained by an underestimation of ETR, as the effect would have been even more prominent.

### Final remarks and future steps

The technique presented here offers a relatively straightforward way to upgrade chamber-based field measurements of photosynthesis, either for short-term campaigns or long-term installations. Long-term and combined measurements of ChlF and gas-exchange can provide a more comprehensive view of the processes underlying the seasonal regulation of photosynthesis, bringing new information to investigate and model the environmental regulation of photosynthesis, such as the temporal dynamics between ChlF and photosynthetic carbon uptake (Gu et al. 2019, Johnson and Berry 2021), which remains a critical step towards the remote sensing of photosynthesis via solar-induced fluorescence (SIF) (Porcar-Castell et al. 2021, Sun et al. 2023).

In the future, the technique could be further improved by (i) replacing the blue PAM measuring light by a red, amber or even green measuring light that penetrates deeper into the leaf (although the signal strength should be checked), to confer a better match between the footprint of the ChlF and gas-exchange measurements (Evans et al. 2017), (ii) improving the synchronization between measurements, which could be attained by controlling all the components from a centralized unit, (iii) integrating a spectrometer system to provide contiguous measurements of leaf spectral reflectance and fluorescence, (iv) adding leaf level temperature measurements for more accurate estimations of VPD and *g_s_*, and last but not least, by (v) complementing the measurements with regular estimations of Abs, α_II_ and the contribution of PSI to total ChlF, in order to provide a more accurate estimate of ETR. In particular, field protocols based on the use of spectral reflectance and fluorescence could be developed, implemented into the setup and used to regularly track variations in the above parameters along ChlF and gas-exchange, providing a truly unique view of the regulation of the photosynthetic apparatus under field conditions.

## Supplementary Material

Supplementary_files_Oivukkamaki_et_al_CORRECTED_PROOF_tpae162

## Data Availability

All primary data (including metadata) within the manuscript are openly available on the Zenodo online repository (doi.org/10.5281/zenodo.13318467).

## Notes

Teemu Hölttä holds the position of Editor for Tree Physiology and has not peer reviewed or made any editorial decisions for this paper.
