# Supplementary material for: Field integration of shoot gas-exchange and leaf chlorophyll fluorescence measurements to study the long-term regulation of photosynthesis in situ
Source: Tree Physiol. 2024 Dec 11;45(1):tpae162. doi: 10.1093/treephys/tpae162 (PMC11775469; doi:10.1093/treephys/tpae162)
Supplement: Supplementary_files_Oivukkamaki_et_al_CORRECTED_PROOF_tpae162 [file supplementary_files_oivukkamaki_et_al_corrected_proof_tpae162.docx]

Figure S1. Filtering out data pairs acquired under contrasting levels of PAR as a result of clock asynchronization. The ratio between cuvette PAR (PAR_CH_) and MICRO-PAM PAR (PAR_MICRO_) was used to discard measurements where A_NET_ and ETR were acquired under contrasting levels of PAR. For the estimation of ETR/A_NET_ we used observation pairs where the ratio was between 0.5 and 2 (marked with the dashed lines in the figure). Points correspond to individual measurements.

Figure S2 Temporal changes in volumetric soil water content during the study period, as measured from 10 cm deep using soil moisture sensors (Delta-T ML3, Delta-T Devices Ltd). Note the gradual decrease in soil water content during July and the effect of the rainfall events, in particular after the second high temperature period (marked with grey shading).





Figure S3 A sudden decrease in MICRO-PAM current F yield was observed during the morning of the 26th July (panel A, black solid line). No similar change in F yield could be observed in the MONI-PAM nearby (black dotted line), suggesting that the drop was possibly caused by a movement of the leaf being measured. To investigate the possible impact of this drop in F on the estimation of the quantum yields of PSII, and although no apparent differences could be observed between the two systems during daytime before and after this drop (panel A, red solid and dashed lines), we compared the relationship between MONI-PAM and MICRO-PAM F_V_/F_M_ for the days before (empty dots) and after (filled dots) the decrease in F (Panel B). The results showed that the slopes were not significantly different from each other (p < 0.05, using an ANOVA test).





Figure S4 Temporal variation of MICRO- and MONI-PAM nighttime F_V_/F_M_ levels during the study period along with daily average temperature. The two high temperature periods are marked with grey shading. Note how MONI-PAM maximum quantum yields of PSII are consistently higher than MICRO-PAM throughout the study period. Data missing after second heat period was caused by an extended power failure.


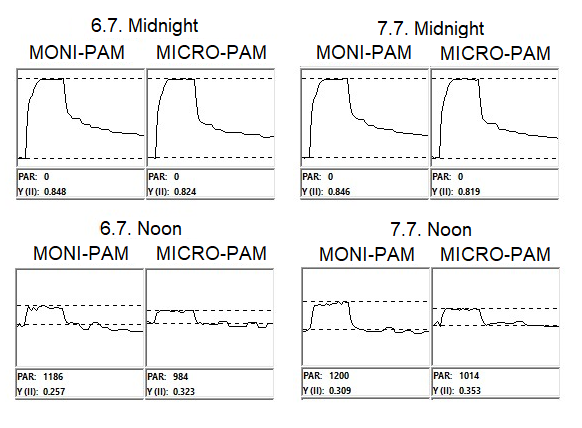


Figure S5 Examples of saturating pulse graphs for the MONI- and MICRO-PAM devices at midnight and noon for two selected days in July. These plots are provided by the WinControl 3.30 Software for diagnostic purposes and reflect the increase in PAM-ChlF from F_S_ to F_M_. In principle, the fact that the F-levels saturate before the end of the pulse would denote that the F_M_ level is reached and that the intensity of the saturating pulse would be sufficiently strong.

<https://blogs.helsinki.fi/optics-of-photosynthesis/files/2022/10/OGES_1.1_short.mp4>

Supplementary video 1. Video presenting a part of the measurement cycle of the combined gas-exchange and ChlF measurements. This video shows the phase of the measuring cycle when the measurements took place. A few seconds before chamber closure, the MICRO-PAM device triggers a saturating pulse and registers F’ and F_M_’ for subsequent estimation of ETR. Next, the chamber is closed and remains in that position for 60 seconds. During this period, air is sampled from the chamber and directed to a gas analyzer in a cottage nearby. Note that the video has been here cut to improve the visualization so that chamber closure time is much shorter than 60 seconds. After measurements, the chamber remains open for 19 minutes so that the full cycle under the current setup was 20 minutes.
